# Supplementary material for: A novel RyR1-selective inhibitor prevents and rescues sudden death in mouse models of malignant hyperthermia and heat stroke
Source: Nat Commun. 2021 Jul 13;12:4293. doi: 10.1038/s41467-021-24644-1 (PMC8277899; doi:10.1038/s41467-021-24644-1)
Supplement: Supplementary file 1 — Supplementary Information [file 41467_2021_24644_MOESM1_ESM.pdf]

Supplementary Information for

# A RyR1-selective inhibitor prevents and rescues sudden death in mouse models of malignant hyperthermia and heat stroke

Toshiko Yamazawa[1†*], Takuya Kobayashi[2†], Nagomi Kurebayashi[2†], Masato Konishi[2], Satoru Noguchi[3], Takayoshi Inoue[4], Yukiko U. Inoue[4], Ichizo Nishino[3], Shuichi Mori[5], Hiroto Iinuma[5], Noriaki Manaka[5], Hiroyuki Kagechika[5], Arkady Uryash[6], Jose Adams[6], Jose R. Lopez[7], Xiaochen Liu[8], Christine Diggle[8], Paul D. Allen[8], Sho Kakizawa[9], Keigo Ikeda[10], Bangzhong Lin[10], Yui Ikemi[10], Kazuto Nunomura[10], Shinsaku Nakagawa[10], Takashi Sakurai[2], Takashi Murayama[2*]

[1]Department of Molecular Physiology, Jikei University School of Medicine, Tokyo, Japan.
[2]Department of Pharmacology, Juntendo University School of Medicine, Tokyo, Japan.
[3]Department of Neuromuscular Research, National Institute of Neuroscience, National Center of Neurology and Psychiatry, Tokyo, Japan.
[4]Department of Biochemistry and Cellular Biology, National Institute of Neuroscience, National Center of Neurology and Psychiatry, Tokyo, Japan.
[5]Institute of Biomaterials and Bioengineering, Tokyo Medical and Dental University, Tokyo, Japan.
[6]Department of Neonatology, Mount Sinai Medical Center, Miami, FL, USA.
[7]Department of Research, Mount Sinai Medical Center, Miami, FL, USA.
[8]Leeds Institute of Biomedical & Clinical Sciences, School of Medicine, University of Leeds, St James's University Hospital, Leeds, UK.
[9]Department of Biological Chemistry, Graduate School of Pharmaceutical Sciences, Kyoto University, Kyoto, Japan
[10]Center for Supporting Drug Discovery and Life Science Research, Graduate School of Pharmaceutical Science, Osaka University, Suita, Japan.
*Address correspondence to: Takashi Murayama (takashim@juntendo.ac.jp), Toshiko Yamazawa (toshiko1998@jikei.ac.jp)

Items contained in this file:

**Supplementary Figures 1-8**

**Supplementary Table 1**

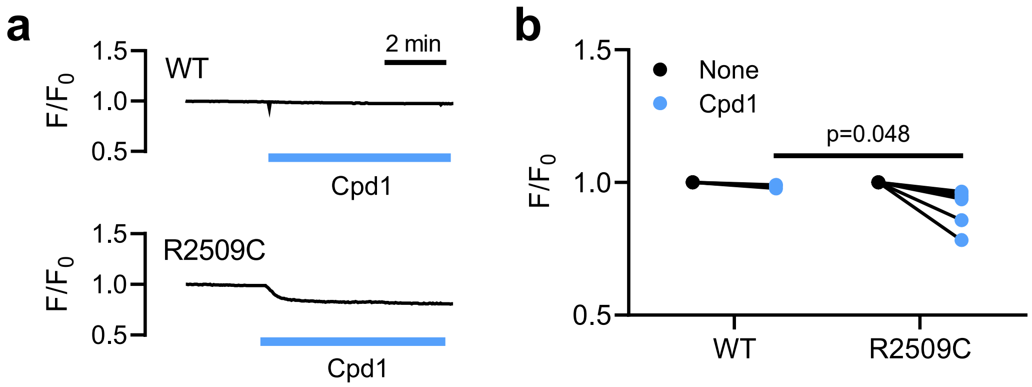


**Supplementary Fig. 1: Effect of Cpd1 on resting $Ca^{2+}$ in the isolated FDB muscle cells from WT and R2509C mice. a** Representative effect of 0.1 μM Cpd1 (blue bar) on resting $Ca^{2+}$ signals in FDB cells. **b** Resting $Ca^{2+}$ levels of WT (*n* = 5) and R2506C (*n* = 6) cells before (black) and during (light blue) application of 0.1 μM Cpd1. Data are shown as means ± SD and were analyzed by two-tailed unpaired *t*-test.

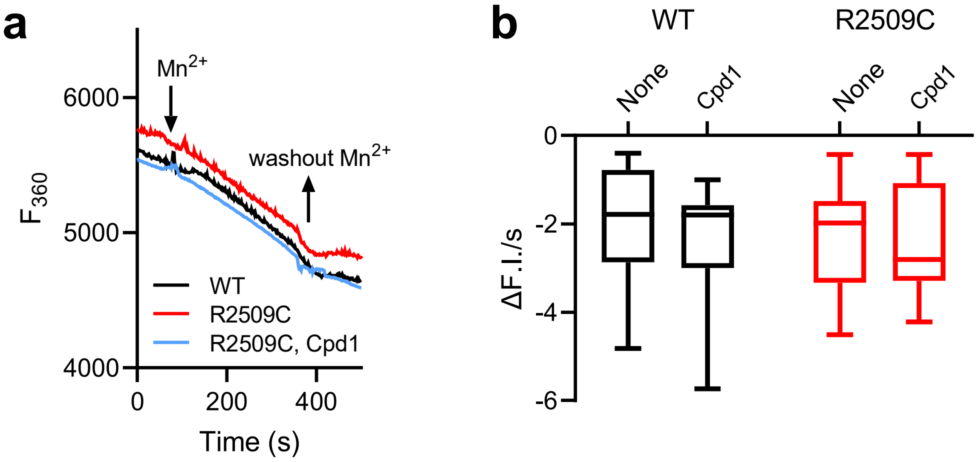


**Supplementary Fig. 2: $Mn^{2+}$ quench assay with the isolated FDB muscle cells from WT and R2509C mice. a** Representative fluorescence declines in fura-2 at 360 nm excitation by $Mn^{2+}$ in the external solution. Slopes for WT (black), R2509C (red) and R2509C with 0.1 μM Cpd1 (light blue) were very similar to each other. **b** Average decline rates of fura-2 fluorescence in the absence and presence of 0.1 μM Cpd1. Data are shown as box-whisker plot, with the median for all subjects shown as the center line, the box representing the 25-75 percentile, and the lines showing the range of the data (WT: *n* = 16; WT, Cpd1: *n* = 14; R2509C: *n* = 18; R2509C, Cpd1: *n* = 12) and were analyzed by two-way ANOVA with Tukey's test. No statistical difference was observed between any groups.

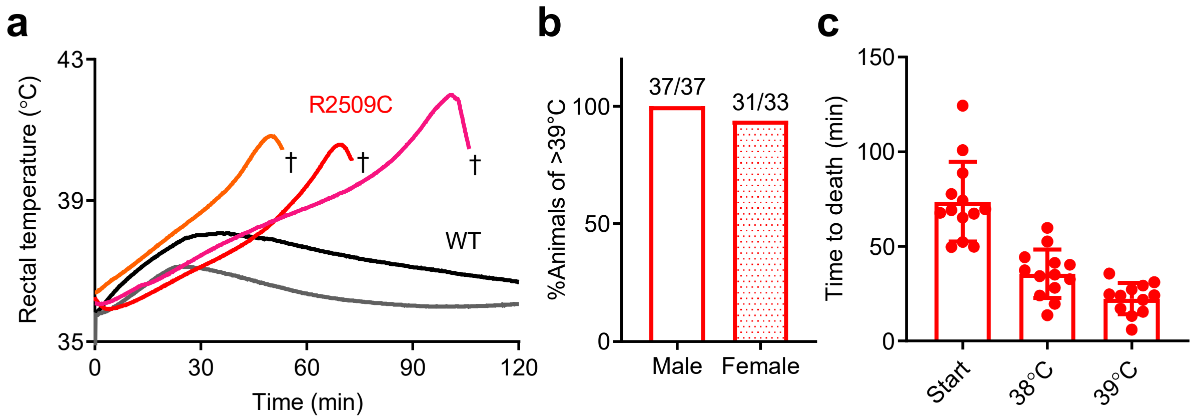


**Supplementary Fig. 3: *In vivo* heat stress challenge of WT and R2509C mice.** Mice were anesthetized and placed in a test chamber at 35°C. **a** Time course of rectal temperature of mice. R2509C mice (colored) but not WT mice (black and grey) exhibited rise in rectal temperature and died by fulminant heat stroke (†). **b** Responsiveness to heat stress. Almost all the mice responded to heat stress. **c** Time to death from start of heat stress challenge or from temperature at 38°C or 39°C. Data are shown as means ± SD (Start: $n$ = 13; 38°C: $n$ = 13; 39°C: $n$ = 12).

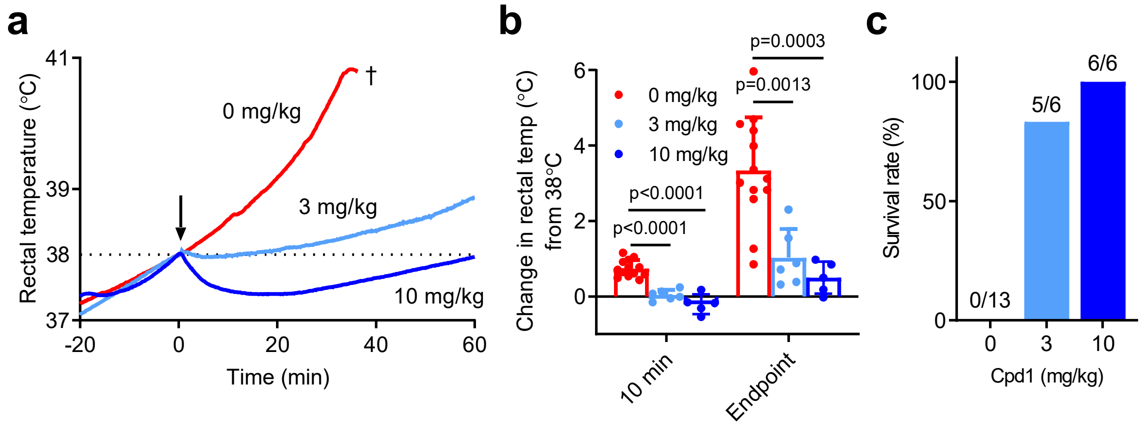


**Supplementary Fig. 4: Rescue effect of Cpd1 on heat stress challenge in R2509C mice.** Cpd1 (0, 3, or 10 mg/kg) was administered i.p. when their body temperature reached 38°C. **a** Time course of rectal temperature in R2509C mice after administration of Cpd1 (arrow) during heat stress challenge. †, death by heat stroke. **b** Change in the rectal temperature 10 min after administration of Cpd1 and the endpoint (60 min after administration or just before death). Data are shown as means ± SD (0 mg/kg: $n$ = 13; 3 mg/kg: $n$ = 6; 10 mg/kg: $n$ = 5) and were analyzed by one-way ANOVA with Tukey's test. **c** Survival rate of mice 60 min after administration of Cpd1.

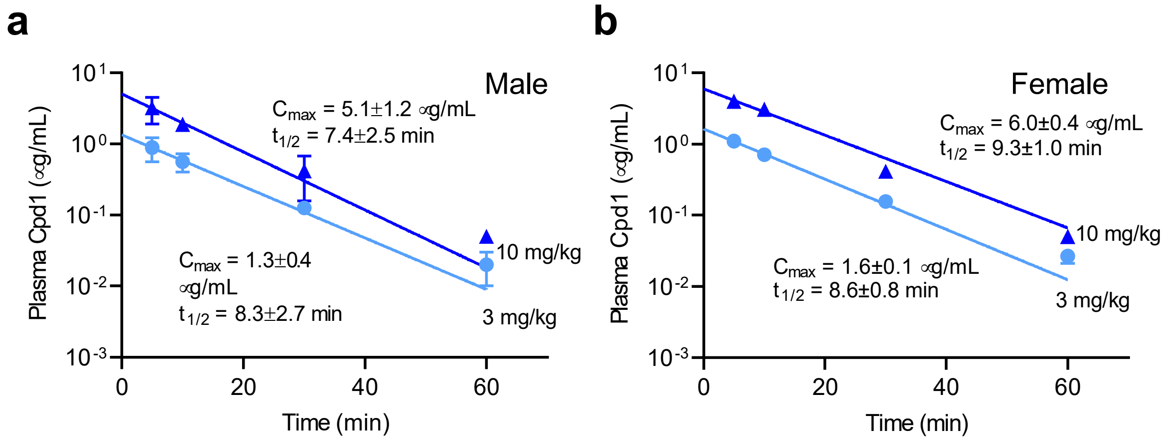


**Supplementary Fig. 5: Pharmacokinetics of Cpd1 in mice**. Average plasma concentration-time profiles of Cpd1 following i.p. injection of 3 mg/kg and 10 mg/kg in male (**a**) and female (**b**) mice. Data are shown as means ± SD (*n* =3 per time point for each group).

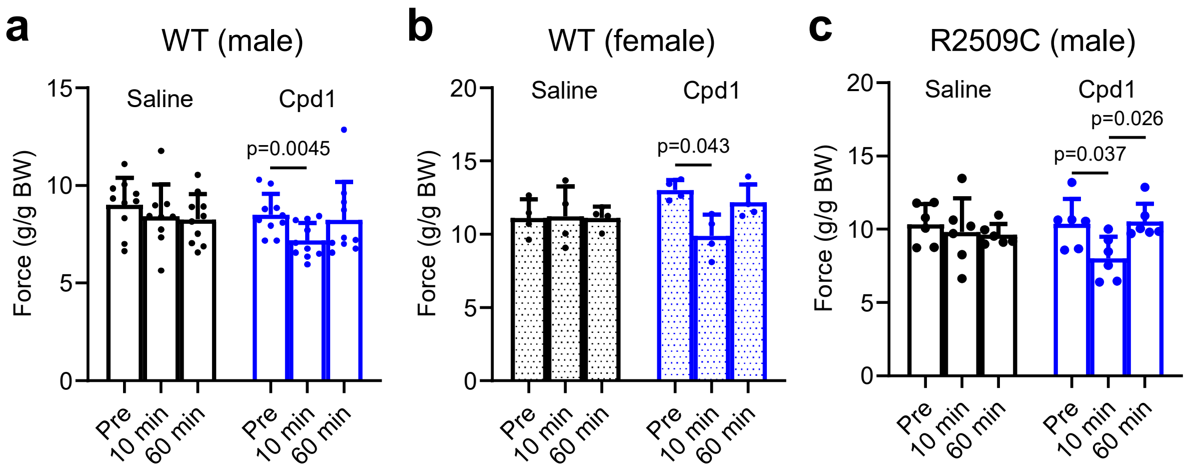


**Supplementary Fig. 6: Effect of Cpd1 on muscle force of WT and R2509C mice.** Muscle force of male WT (**a**), female WT (**b**) and male R2509C (**c**) mice was measured *in vivo* by grip force test (4-grips test). The tests were performed before (Pre) and 10 and 60 min after i.p. injection of saline or Cpd1. The force values were normalized by body weight (BW). Data are shown as means ± SD (male WT: *n* = 10; female WT: *n* = 4; male R2509C: *n* = 6) and were analyzed by one-way ANOVA with Tukey's test.

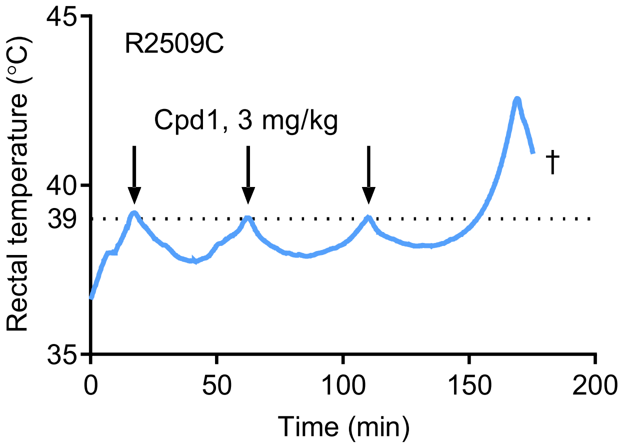


**Supplementary Fig. 7: Effect of repeated application of Cpd1 on *in vivo* isoflurane challenge of R2509C mouse.** Rectal temperature in male R2509C mouse was measured after anesthesia by isoflurane. Cpd1 (3 mg/kg) was repeatedly administered i.p. when the body temperature reached 39°C (arrow). †, death by fulminant MH crisis.

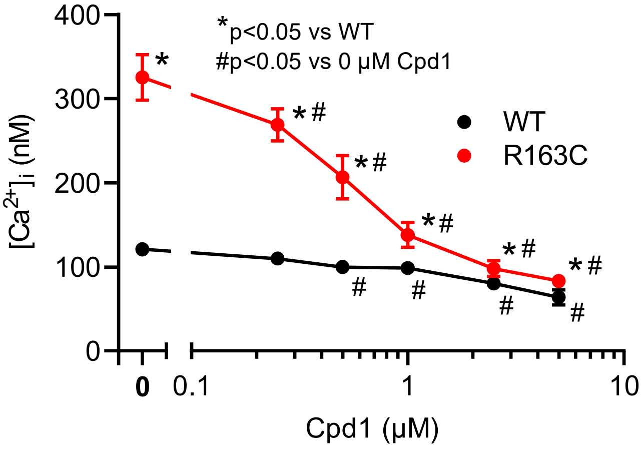


**Supplementary Fig. 8: *In vitro* $[Ca^{2+}]_i$ homeostasis of skeletal muscles isolated from WT and R163C mice.** FDB muscle fibers were isolated and $[Ca^{2+}]_i$ was determined using $Ca^{2+}$ selective microelectrodes. $[Ca^{2+}]_i$ was significantly higher in quiescent R163C fibers (326 ± 27 nM, *n* = 16) compared to WT fibers (121 ± 3 nM, *n* = 20). Cpd1 reduced $[Ca^{2+}]_i$ in a dose-dependent manner. Data are shown as means ± SD (0 µM: *n* = 20; 0.25 µM: *n* = 13; 0.5 µM: *n* = 15; 1 µM: *n* = 11; 2.5 µM: *n* = 13; 5 µM: *n* = 10 for WT and 0 µM: *n* = 16; 0.25 µM: *n* = 13; 0.5 µM: *n* = 14; 1 µM: *n* = 13; 2.5 µM: *n* = 15; 5 µM: *n* = 13 for R163C) and were analyzed by two-way ANOVA with Tukey's test.

**Supplementary Table 1. The primers used in this study.**

| Name | Sequence |
|---|---|
| R2509C_Forward | GCGTCAGGAATGGTTAGAATAGAT |
| R2509C_Reverse | TTGTCTGATGCTAGGTAGAAGGTG |
