# Supplementary material for: A novel RyR1-selective inhibitor prevents and rescues sudden death in mouse models of malignant hyperthermia and heat stroke
Source: Nat Commun. 2021 Jul 13;12:4293. doi: 10.1038/s41467-021-24644-1 (PMC8277899; doi:10.1038/s41467-021-24644-1)
Supplement: Supplementary file 2 — Reporting Summary [file 41467_2021_24644_MOESM2_ESM.pdf]

nature research

Corresponding author(s): Takashi Murayama

Last updated by author(s): May 14, 2021

Reporting Summary

Nature Research wishes to improve the reproducibility of the work that we publish. This form provides structure for consistency and transparency in reporting. For further information on Nature Research policies, see our Editorial Policies and the Editorial Policy Checklist.

Statistics

For all statistical analyses, confirm that the following items are present in the figure legend, table legend, main text, or Methods section.

n/a	Confirmed	
☐	☒	The exact sample size (n) for each experimental group/condition, given as a discrete number and unit of measurement
☐	☒	A statement on whether measurements were taken from distinct samples or whether the same sample was measured repeatedly
☐	☒	The statistical test(s) used AND whether they are one- or two-sided<br>*Only common tests should be described solely by name; describe more complex techniques in the Methods section.*
☒	☐	A description of all covariates tested
☐	☒	A description of any assumptions or corrections, such as tests of normality and adjustment for multiple comparisons
☐	☒	A full description of the statistical parameters including central tendency (e.g. means) or other basic estimates (e.g. regression coefficient) AND variation (e.g. standard deviation) or associated estimates of uncertainty (e.g. confidence intervals)
☐	☒	For null hypothesis testing, the test statistic (e.g. F, t, r) with confidence intervals, effect sizes, degrees of freedom and P value noted<br>*Give P values as exact values whenever suitable.*
☒	☐	For Bayesian analysis, information on the choice of priors and Markov chain Monte Carlo settings
☒	☐	For hierarchical and complex designs, identification of the appropriate level for tests and full reporting of outcomes
☒	☐	Estimates of effect sizes (e.g. Cohen's d, Pearson's r), indicating how they were calculated

Our web collection on statistics for biologists contains articles on many of the points above.

Software and code

Policy information about availability of computer code

Data collection	Metamorph (v7, MolecularDevices)<br>LabChart (v6, ADInstruments)<br>Piper (v1.3.04, Stanford Photonics).
Data analysis	GraphPad Prism (v7 and v8)

For manuscripts utilizing custom algorithms or software that are central to the research but not yet described in published literature, software must be made available to editors and reviewers. We strongly encourage code deposition in a community repository (e.g. GitHub). See the Nature Research guidelines for submitting code & software for further information.

Data

Policy information about availability of data

All manuscripts must include a data availability statement. This statement should provide the following information, where applicable:

- Accession codes, unique identifiers, or web links for publicly available datasets
- A list of figures that have associated raw data
- A description of any restrictions on data availability

All data generated or analyzed in this study are available within the article and its Supplementary Information. All raw data supporting the findings from this study are available from the corresponding author upon request. Source data are provided with this paper.

Field-specific reporting

Please select the one below that is the best fit for your research. If you are not sure, read the appropriate sections before making your selection.

- [x] Life sciences
- [ ] Behavioural & social sciences
- [ ] Ecological, evolutionary & environmental sciences

For a reference copy of the document with all sections, see nature.com/documents/nr-reporting-summary-flat.pdf

Life sciences study design

All studies must disclose on these points even when the disclosure is negative.

Sample size	The sample size for each experiment was determined based on pilot experiments, which provided an estimate of the effect size and of data variability.
Data exclusions	No data were excluded in this study.
Replication	All in vitro and animal experiments were repeated at least three times independently over the past two years. All attempts at replication were successful and produced similar results.
Randomization	In this study, randomization was not performed, because allocation was not performed.
Blinding	Blinding was performed to group allocation for measurement of muscle force in vivo (Figs 6c, 6h, S6a-c). The other in vivo studies which measure rectal temperature (Figs 1, 4, 5 and 8) or cytoplasmic Ca2+ (Fig. 7) was not blinded, since the risk of bias is low.

Reporting for specific materials, systems and methods

We require information from authors about some types of materials, experimental systems and methods used in many studies. Here, indicate whether each material, system or method listed is relevant to your study. If you are not sure if a list item applies to your research, read the appropriate section before selecting a response.

Materials & experimental systems

n/a	Involved in the study	
x		Antibodies
x		Eukaryotic cell lines
x		Palaeontology and archaeology
	x	Animals and other organisms
x		Human research participants
x		Clinical data
x		Dual use research of concern

Methods

n/a	Involved in the study	
x		ChIP-seq
x		Flow cytometry
x		MRI-based neuroimaging

Animals and other organisms

Policy information about studies involving animals; ARRIVE guidelines recommended for reporting animal research

Laboratory animals	heterozygous RYR1-p.R2509C mice (C57BL/6 background, 8-20-week old), heterozygous RYR1-p.163C mice (C57BL/6 background, 12-16-week old), homozygous RYR1-p.G2435R mice (C57BL/6 background, 12-16-week old). Housing conditions are described in Methods.
Wild animals	No wild animals were used in this study.
Field-collected samples	This study did not involve samples collected from the field.
Ethics oversight	Animal experiments were approved and were in accordance with the guidelines of the Jikei University School of Medicine, Juntendo University School of Medicine, the National Center of Neurology and Psychiatry (NCNP), Mount Sinai Hospital and Leeds University.

Note that full information on the approval of the study protocol must also be provided in the manuscript.
